# Gut microbiome analysis by post: Evaluation of the optimal method to collect stool samples from infants within a national cohort study

**DOI:** 10.1371/journal.pone.0216557

**Published:** 2019-06-12

**Authors:** Georgina M. Williams, Sam D. Leary, Nadim J. Ajami, Saranna Chipper Keating, Joseph F. Petrosin, Julian P. Hamilton-Shield, Kathleen M. Gillespie

**Affiliations:** 1 The NIHR Bristol Biomedical Research Centre, Nutrition Theme, University of Bristol, Bristol, United Kingdom; 2 Diabetes and Metabolism, Bristol Medical School, University of Bristol, Bristol, United Kingdom; 3 Alkek Center for Metagenomics and Microbiome Research, Department of Molecular Virology and Microbiology, Baylor College of Medicine, Houston, Texas, United States of America; Fred Hutchinson Cancer Research Center, UNITED STATES

## Abstract

**Background:**

Understanding the role of the gut microbiome is pivotal for the future development of therapies for the prevention and management of autoimmune conditions such as type 1 diabetes when sampling during early life may be particularly important. The current standard methods for collecting gut microbiome samples for research is to extract fresh samples or freeze samples immediately after collection. This is often impractical however for population-based studies. The aim of this study was to determine the optimal method for the stabilization of stool bacterial DNA obtained from nappies and transported by post in ambient conditions to the research centre for a national birth cohort study.

**Methods:**

Four methods to collect samples were compared to immediate freezing of samples: 1) collecting faeces onto a swab which was immediately frozen, 2) using a commercially available kit with stabilisation solution (OMNIgene•GUT kit) at ambient temperature, 3) collecting onto a swab and 4) collecting into a sterile plain tube. Samples 3) and 4) were returned to the laboratory by post at ambient temperatures. A Bland Altman analysis was used to assess the agreement between the different methods and the frozen standard.

**Results:**

Stool samples were collected by parents. For samples transported in ambient conditions, the limits of agreement showed that the OMNIgene•GUT kit had the narrowest 95% limits of agreement with the frozen standard as measured by the number of operational taxonomic units and the Shannon diversity index.

**Conclusions:**

All methods assessed for preserving samples collected from nappies at a distance and delivered by post for gut microbiome analysis showed variation / disagreement from the frozen standard. Overall, the OMNIgene•GUT kit preserved the samples with minimal changes compared to other methods and was practical for parents to use.

## Background

The human gut microbiome consists of trillions of bacterial cells. As evidence for its underlying role in health and disease emerges, interest in its constituents and function are increasing [1, 2]. Microbial colonization of the gastrointestinal tract during the first few years of life appears to be essential for future host immune regulation. Perturbations in the composition or function of the microbiota has been associated with a variety of inflammatory and autoimmune conditions [2–5]. Although studies are still preliminary, there are indications that children from countries where levels of autoimmunity are high, have increased levels of *Bacteroides* species in their gut microbiota which may act to disrupt the development of immune tolerance to self-antigens although the underlying mechanisms are unclear [2]. Type 1 diabetes appears to be associated with a gut microbiome that is less diverse than the non-diabetic population [4, 6, 7]. In order to identify distinguishing features of the gut microbiome that vary between individuals and in different conditions, accurate sampling methods are essential. Faecal samples have been shown to be representative of the distal intestinal microbiome [8]. Freezing the stool sample immediately preserves the sample for gut microbiome analysis [9] but is impractical and/or too expensive for many large studies. Standard methods for self-collection (or for parental collection of samples from nappies) which ensure samples are stabilized during transport at ambient temperatures are required.

Children with Down’s Syndrome are at increased risk of developing autoimmune conditions very early in life [10]. Feeding and Autoimmunity in Down’s Syndrome Evaluation Study (FADES) was established to determine the feasibility of creating a national cohort of infants with Down’s syndrome (DS) to study associations between early infant feeding, infections, the gut microbiome and the development of autoimmunity. As part of the operational development of the FADES study (www.uhbristol.nhs.uk/research-innovation/our-research/bristol-nutrition-bru/fades-study/) the optimal method to collect gut microbiome samples by post from infants was examined. The aim of this study therefore was to determine the optimal method to preserve the integrity of gut microbiome samples during collection and transport to the laboratory by post.

## Material and methods

Initially 10 participants were recruited under the age of 1 year (6 males / 4 females; age range 6 to 38 weeks) who had been admitted to Bristol Royal Hospital for Children without diarrhoeal disease. Ethical approval was obtained from the South West Central Bristol Research Ethics Committee (14/SW/0030). Parents were asked to check their child’s nappy regularly and let the researchers know as soon as they identified their infant had a dirty nappy to ensure samples were fresh.

From a single stool (one “dirty” nappy) from each participant, samples were collected as follows: a stool sample into 1) an empty sterile tube (plain tube), 2) a swab, both of which were immediately frozen on dry ice. Then samples were collected into a 3) plain tube 4) onto a swab and finally 5) into the OMNIgene•GUT stool stabilisation fluid. The OMNIgene•GUT kit (commercially available from DNA Genotek Inc. Ottawa, ON, Canada) consists of a tube of stabilisation liquid and a ball bearing. The faeces are placed into the tube lid which is designed to break up the faeces and the ball homogenises the sample when it is shaken. Samples 3)-5) were posted to the Diabetes and Metabolism Unit at Southmead Hospital, Bristol, UK.

All samples returned in the post (ambient conditions) arrived within 3 days of sampling. Our laboratory routinely collects capillary blood and mouth brush samples by post. We ask participants to take and post the samples on Sunday-Tues to ensure that they spend the minimum time possible in the post. On arrival the samples were frozen immediately at -80⁰C. Samples (n = 48) were shipped frozen by courier to The Alkek Center for Metagenomics and Microbiome Research (CMMR), at Baylor College of Medicine, Houston, Texas. There were nine participants who provided sufficient stool for all five sample types and one child who only provided sufficient sample for the OMNIgene Gut kit and two swabs (one at room temperature and one frozen).

The samples were subjected to extraction for 16S rRNA gene profiling using gene sequencing methods adapted from those developed for the Earth Microbiome Project [11, 12] and the NIH-Human Microbiome Project [13, 14]. Briefly, bacterial genomic DNA was extracted using MO BIO PowerSoil DNA Isolation Kit (MO BIO Laboratories) following the manufacturer’s instructions. The 16S rRNA gene V4 region was amplified by PCR and sequenced in the MiSeq platform (Illumina) using the 2x250 bp paired-end protocol yielding pair-end reads. The primers used for amplification contain adapters for MiSeq sequencing and single-end barcodes allowing pooling and direct sequencing of PCR products [12].

The 16S rRNA gene pipeline data incorporates phylogenetic and alignment-based approaches to maximize data resolution [15]. The read pairs were demultiplexed based on the unique molecular barcodes, and reads were merged using USEARCH v7.0.1090 [16], allowing zero mismatches and a minimum overlap of 50 bases. Merged reads were trimmed at the first base with Q5. In addition, a quality filter was applied to the resulting merged reads and those containing above 0.05 expected errors were discarded.

16Sv4 rRNA gene sequences were clustered into operational taxonomic units (OTUs) at a similarity cut-off value of 97% using the UPARSE algorithm. OTUs were mapped to an optimized version of the SILVA 123 Database [16, 17] containing only the 16S v4 region to determine taxonomies. Abundances were recovered by mapping the demultiplexed reads to the UPARSE OTUs. A custom script constructed a rarefied OTU table from the output files generated in the previous two steps for downstream analyses of taxonomic relative abundance, alpha-diversity (number of OTUs–richness and Shannon’s diversity index), beta-diversity (including UniFrac) [18], and phylogenetic trends. The 10 most abundant Phyla, Classes, Order, Families and Genera observed in the 48 samples analysed are shown in S1 Fig.

### Statistical methods

To assess the changes seen in diversity of the samples, comparisons were made of the number of OTUs which represent the array of species observed. To compare whether changes were related to the richness and evenness of the microbes within samples, the Shannon diversity index (Shannon index) was used. For each stool sample, the difference in the measurement (number of OTU’s or Shannon index) between the frozen standard and each of the methods of sample collection were plotted against the mean of the two measurements (a Bland-Altman (BA) plot) [19]. The raw data are available in S1 Data. This test was used because we wished to measure the agreement between the frozen standard and the practical sample collection alternatives. The differences and the means were unrelated and therefore 95% limits of agreement could be calculated (mean difference +/- 2 standard deviations of the difference) and added to the plot. This gave the range of disagreement between the frozen standard and each of the sampling methods [19].

The relative abundance of each of the four major phyla (*Actinobacteria*, *Bacteroidetes*, *Firmicutes* and *Proteobacteria;* (other phyla were rare in these samples–S1 Fig) and the ten main genera (*Acinetobacter*, *Bifidobacterium*, *Enterobacter*, *Esherichia_Shigella*, *Lactobacillus*, *Peptoclostridium*, *Staphylococcus*, *Streptococcus*, *Subdoligranulum* and *Veillonella*) were calculated and plotted on a stacked bar chart (Microsoft Excel) to allow a visual comparison. Limits of agreement were calculated and used to compare the abundance of each of the four main phlya between the frozen standard and the other sampling methods. The same analysis was also completed for *Enterobacter*, *Escherichia Shigella*, *Bifidobacterium*, *Streptococcus*, *Staphylococcus*, *Veillonella*, *Lactobacillus* and *Rothia*. These are all genera which were present in the samples and in the microbiota have been associated with type 1 diabetes auto-antibody seropositivity, breastfeeding or other dietary components [2].

## Results

### Recruitment

The initial recruitment target was for 10 participants. One of the infants recruited did not produce an adequate stool volume to test all sampling methods (sufficient sample was available only for 3 tests; the Genotek method and frozen/posted swab). Analysis of initial profiling (S1 Fig) showed that one child had a significant *Clostridium* infection and the samples were removed from further analysis.

The Bland Altman analysis is therefore presented on data from 8 infants (6 males / 2 females; age range 6 to 38 weeks) with five different sampling methods.

### Run metrics

There were 516,160 reads across the initial 48 samples tested with a median of 10,781 reads. Rarefaction is a process of normalizing all samples to the same total amount of reads. The rarefied read number for each sample was 6,170.

### Impact of sampling method on community diversity

**Fig 1:**
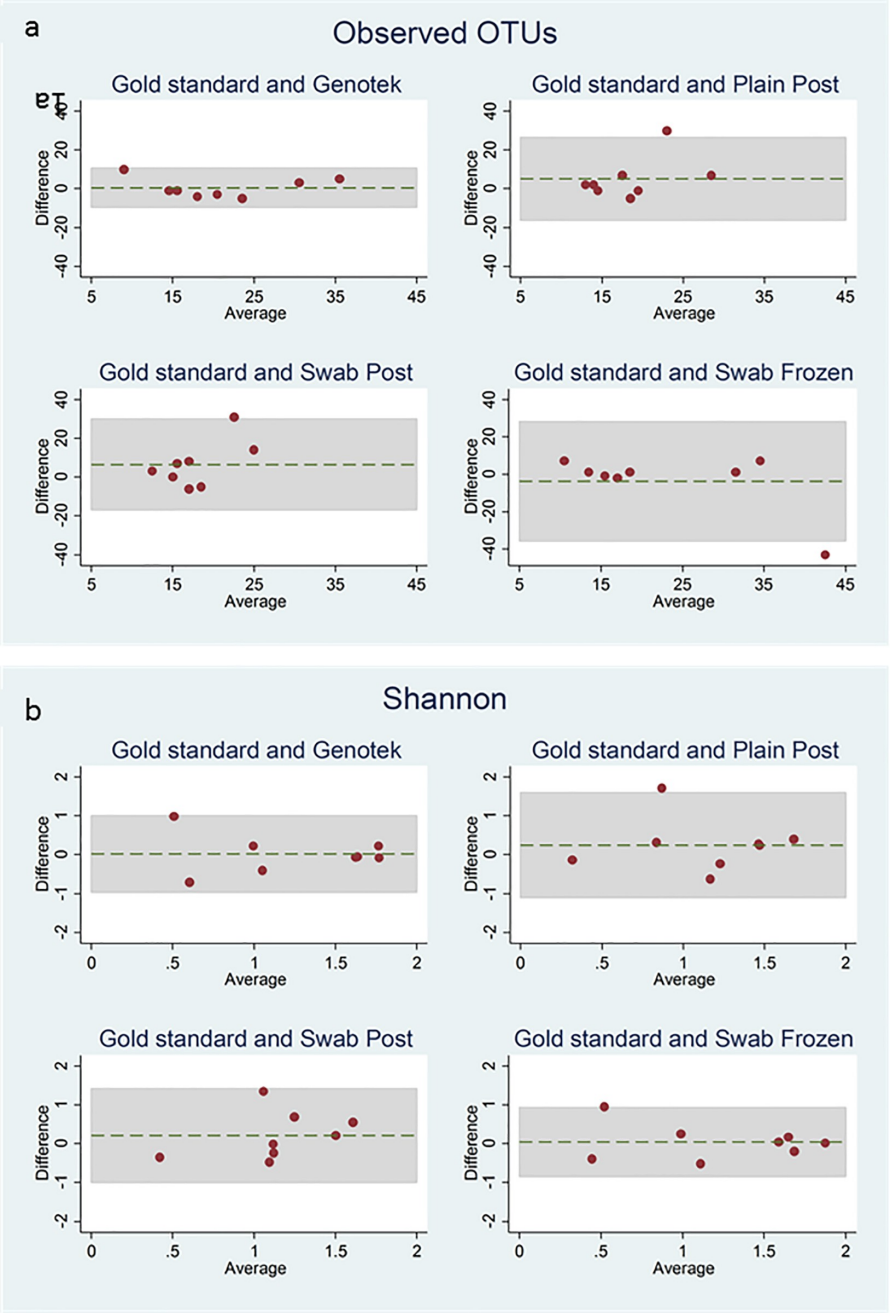
**a) and b)**. Bland Altman plots of observed OTUs (1a) and Shannon index (1b) for the four different methods of sample collection compared to the frozen standard of immediate freezing.

The BA analysis of the observed OTUs (Table 1, Fig 1A) showed the OMNIgene•GUT kit to have the narrowest limits of agreement (-9.55, 10.55) with the frozen standard. The limits of agreement for the samples sent in ambient conditions (plain post and swab post) with the frozen standard, were comparable with each other as expected (-16.13, 26.38 and -16.95, 29.95), and showed less agreement with the frozen standard than the OMNIgene•GUT kit. The frozen swab compared to the frozen standard showed less agreement than would be expected (-35.49, 28.24) due to a single outlier.

**Table 1:** Methodology compared to frozen standard (average difference, (95% limits of agreement)).

	
	OMNIgene•GUT	Plain post	Swab post	Swab frozen
**OTU**	0.50 (-9.55, 10.55)	5.13 (-16.13, 26.38)	6.50 (-16.95, 29.95)	-3.63 (-35.49, 28.24)
**Shannon Index**	0.02 (-0.96, 1.00)	0.25 (-1.10, 1.59)	0.21 (-0.99, 1.42)	0.04 (-0.85, 0.93)

Bland Altman analysis of OTU and Shannon index for the four different methods of sample collection compared to the standard of immediate freezing

The BA analysis comparing the Shannon index results (Table 1, Fig 1B) showed that for all methods, the limits of agreement were narrow suggesting that Shannon index is not affected greatly by collection method or by being exposed to ambient temperature. The closest agreement with the frozen standard was seen in the samples which were collected using a swab and frozen immediately (-0.85,0.93). For samples taken and exposed to ambient conditions in the post the OMNIgene•GUT kit showed the closest agreement to the frozen standard method (-0.96, 1.0), with the plain post and swab post samples showing the least agreement as seen with analysis of OTUs (-1.1, 1.59 and -0.99, 1.42).

### Impact of sampling methods on community composition

**Fig 2:**
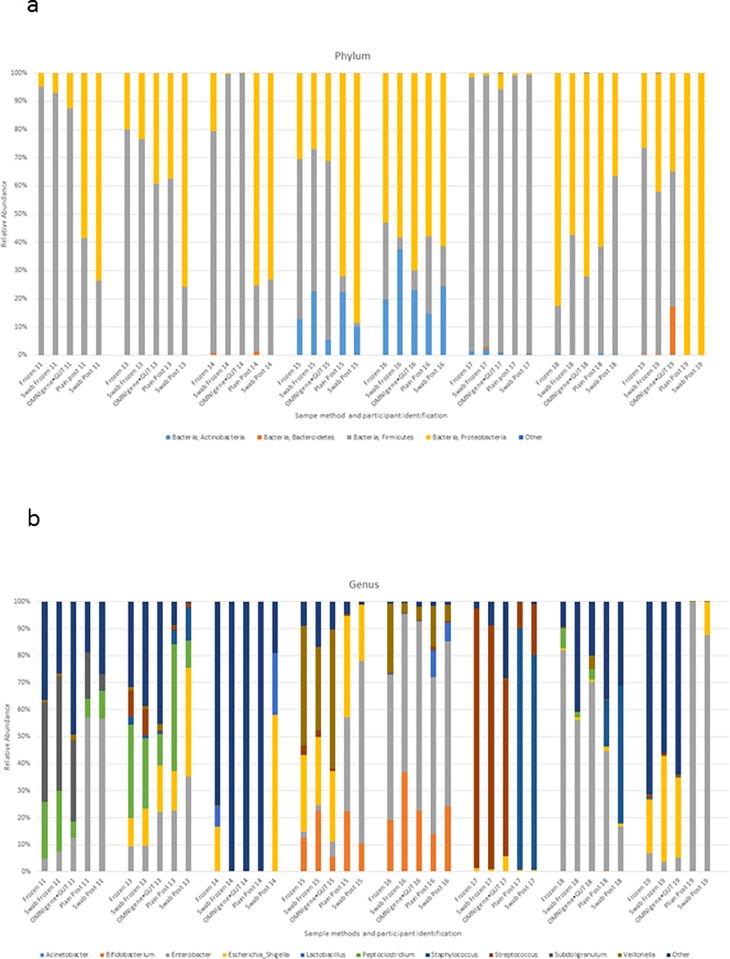
**a)** Relative abundance of the four main phyla for each sampling method (immediate freezing in a plain sterile tube (frozen), immediate freezing on a swab (swab frozen), using the OMNIgene•GUT kit (OMNIgene•GUT), collecting into a plain sterile tube in the post (plain post) and collecting onto a swab which is sent in the post (swab post). **b)** Relative Abundance of the ten main genera for each sampling method.

As would be expected when samples are subjected to different conditions during sampling and transport the relative abundance of the phyla changed as the conditions either encouraged the proliferation or suppressed the growth of microbes. The results presented are for the four phyla which were the most abundant within the infant gut microbiome, these are *Firmicutes*, *Proteobacteria*, *Actinobacteria* and *Bacteroidete*s (S1 Fig). Fig 2A shows the variation in the relative abundance of different Phyla between individuals. Each of these infants appears to have a distinct gut microbiome. For five of the participants (ID 11, 14, 15, 18 and 19) the profile seen in the samples collected using the OMNIgene•GUT kit resembles the frozen standard sample method of collecting into a plain tube and freezing immediately. There has been an overgrowth of *Proteobacteria* within the samples exposed to ambient temperatures with a relative suppression of *Firmicutes*.

The limits of agreement (Table 2, S2A and S2B Fig) for the relative abundance of *Firmicutes* and *Proteobacteria* showed the OMNIgene•GUT kit and the frozen swab to show the closest agreement with the frozen standard method. The plain tube and the swab sent in the post showed that changes had occurred in the abundances of these phyla.

**Table 2:** Methodology compared to frozen standard (average difference, (95% limit of agreement).

Phylum	OMNIgene•GUT	Plain post	Swab post	Swab frozen
***Actinobacteria***	35.6(-320.5, 391.7)	-24.6(-522.2, 473)	-6.3 (-249.2, 236.7)	-213.4(-1019.8, 593)
***Bacteroidetes***	-124(-851.8, 603.8)	-3.3(-30.7, 24.2)	6.8(-24.6, 38.1)	-0.2(-47.4, 46.9)
***Firmicutes***	288(-1729.2, 2305.2)	1755.5(-2379.7, 5890.7)	2081(-2960.2, 7122.7)	34.8(-1966.1, 2035.6)
***Proteobacteria***	-198.4 (-1822.3, 1425.6)	-1727.5 (-5683.6, 2228.6)	-2081.4 (7182.5, 3019.8)	180.8 (-1452.2, 1813.7

Bland Altman analysis of the relative abundance of the four most abundant phyla for the four different methods of sample collection compared to the gold standard of immediate freezing.

The BA plots for *Acinetobacteria* (S2C Fig) show that the growth of *Acinetobacteria* is relatively unaffected by collecting samples into a plain tube or swab and transporting in ambient conditions than from using the OMNIgene•GUT kit. The frozen swab samples showed a large disagreement from the frozen standard method which is unexpected. The agreement in the amount of *Bacteroidetes* seen between the frozen standard and the other sampling methods was comparable for all apart from the OMNIgene•GUT kit (S2D Fig). This had a single outlier affecting the result, if this were to be removed (but it was not removed), all of the sampling methods would show a similar agreement with the frozen standard method.

The variation in the relative abundance of the different genera within the gut microbiome is much greater between individual infants than it is between the sampling methods (Fig 2B). The method of collection and exposure to ambient temperatures does affect, to a variable degree, the growth or suppression of different genera (Table 3, Fig 2B).

**Table 3:** Methodology compared to frozen standard (average difference, (95% limit of agreement).

Genus	OMNIgene•GUT	Plain post	Swab post	Swab frozen
***Enterobacter***	-206.6 (-1271.6, 858.4)	-1217 (-6025.2, 3590.7)	-1266.9 (-6866.4, 4332.6)	162.4 (-982.2, 1307.0)
***Staphylococcus***	19.4 (-65.1, 103.9)	-848.3 (-4631.4, 2934.9)	-1066.4 (-4761.4, 2628.6)	17.3 (-54.4, 88.9)
***Bifidobacterium***	31.0 (-325.7, 387.7)	-32.8 (-538.1, 472.6)	-15.9 (-252.3, 220.5)	-211.6 (-1029.0, 605.7)
***Lactobacillus***	60.88 (-275.8, 397.6)	-16.6 (-600.9, 567.7)	-170.5 (-853.1, 512.1)	61.8 (-274.3, 397.8)

Bland Altman analysis of the relative abundance of *Enterobacter*, *Staphylococcus*, *Lactobacillus* and *Bifidobacterium* for the four different methods of sample collection compared to the gold standard of immediate freezing.

To explore the variations in the abundance of the genera between sampling methods limits of agreement for four genera are shown (S3A, S3B, S3C and S3D Fig). *Enterobacter* and *Staphylococcus* were chosen due to the large difference in agreement seen between sampling methods. *Bifidobacterium* and *Lactobacillus* are both linked to autoimmunity and are affected by feeding methods and therefore are particularly relevant for the collection of samples for FADES.

Both for *Enterobacter* and *Staphylococcus* there was relatively close agreement between the frozen standard method and collecting samples onto a swab which was immediately frozen, as might be expected. A similarly close agreement was seen for the samples collected using the OMNIgene•GUT kit. For both *Enterobacter* and *Staphylococcus*, the samples exposed to ambient temperatures showed a distinct abundance of these bacteria from the frozen standard. These differences, although less marked, were also similar for *Lactobacillus*. For *Bifidobacterium* the limits of agreement were similar to those observed for the phyla *Actinobacteria* as *Bifidobacterium* is a member of the *Actinobacteria* phylum and the differences seen in both S2C and S2D Fig) are due to differences in growth of *Bifidobacterium* between the sampling methods.

## Discussion

We have shown that there is variation in microbiome results from four different collection methods compared with the frozen standard of immediate freezing. This study demonstrates that the OMNIgene•GUT kit is straightforward to use and preserves samples taken from nappies and exposed to ambient temperatures. The OMNIgene•GUT kit can therefore be considered the best practical option for distance sample collection in general population studies.

Although parents were asked to check the baby’s nappy regularly we do not know how fresh the stool samples were although they were all likely to have been collected within 2 hours of having been passed. Samples sent through the post took three days to arrive in the laboratory. Sample collection at home for population-based studies may result in samples being exposed to ambient temperatures for longer periods of time although our laboratory protocols try to overcome this by asking participants to post samples on Monday or Tuesday to arrive in the laboratory before the weekend. The OMNIgene•GUT kit has recently been shown to preserve samples at ambient temperatures for up to 60 days [20, 21].

This is the first methodological study defining the method of choice for infants less than one year of age. Although a relatively small study, all sampling methods were consistent, each method was tested within all eight participants samples and the genus data demonstrated large inter-individual variation. The infants analysed in this study appeared to each have distinctive microbiomes.

A recent ground breaking study of the longitudinal gut microbiome in children participating in the Environmental Determinants of Diabetes in the Young analysed using the same laboratory and methods used in this study, showed that the developing gut microbiome undergoes three distinct phases of microbiome progression: a developmental phase (months 3–14), a transitional phase (months 15–30), and a stable phase (months 31–46) [22]. Receipt of breast milk, either exclusive or partial, was the most significant factor associated with the microbiome structure. Breastfeeding was associated with higher levels of *Bifidobacterium* species (*B*. *breve* and *B*. *bifidum*), and the cessation of breast milk resulted in faster maturation of the gut microbiome, as marked by the phylum *Firmicutes*. Birth mode was also significantly associated with the microbiome during the developmental phase, driven by higher levels of *Bacteroides* species (particularly *B*. *fragilis*) in infants delivered vaginally. *Bacteroides* was also associated with increased gut diversity and faster maturation, regardless of the birth mode.

The amount of faecal material that it is possible to collect from nappies depends on the baby’s age and feeding methods. Very liquid stools are rapidly absorbed into the nappy. For each of the methods described in this paper very little faecal matter was required. A previous study to compare collection methods from young children and the elderly also showed the OMNIgene•GUT to perform well at ambient temperatures for children with a median age of 2 years old [20] and for collection of samples from adults [23].

Previous studies comparing sampling and storage methods for microbiome samples have used measures of dissimilarity and correlation such as Bray-Curtis and Wilcoxon signed rank, paired DESeq, Mann-Whitney-Wilcoxon and Kruskal-Wallis tests [20, 24, 25]. These show degrees of association between the sampling methods rather than an agreement. In this study, we conducted a Bland Altman analysis allowing analysis of agreement between each collection method with the frozen “gold” standard; this is a novel application of this statistical method for comparison of microbiome methodologies.

This study as with others, shows that for all collection methods there is a change in the gut microbiome samples once they are exposed to ambient temperatures. In particular, there was an overgrowth of *Proteobacteria* and a relative reduction in abundance of *Firmicutes* in those samples exposed to ambient conditions. However, it also demonstrates that the OMNIgene•GUT kit preserves the samples with the least change to the diversity of the species seen (number of OTUs) or the richness and evenness of the samples (Shannon index).

This approach is now being used to collect all gut microbiome samples from FADES participants and other cohorts from the general population.

## Conclusions

This paper establishes a workable standard protocol for the collection of stool samples for birth cohorts such as FADES and for larger population studies involving adults and children where geographical restraints and costs mean that the immediate freezing of stool samples for gut microbiome analysis is not possible. It shows gut microbiome samples from infants are best preserved when using the OMNIgene•GUT kit compared to collecting onto a swab or into a plain sterile pot and sending in the post.

## Supporting information

S1 Fig The top ten phyla, classes, orders and families of bacteria detected in the initial analysis of 48 samples before BA analysis. (TIF)

S2 Fig The Bland–Altman plots for abundance of a)*Firmicutes*, b)*Proteobacteria*, c)*Actinobacteria* and d)*Bactriodetes* for the four different methods of sample collection compared to the frozen standard of immediate freezing.
(TIF)

S3 Fig The Bland–Altman plots for abundance of a)*Enterobacter*, *b)Bifidobacterium*, *c)Staphylococcus* and *d)Lactobacillus* for the four different methods of sample collection compared to the frozen standard of immediate freezing.
(TIF)

S1 Data Raw data file including reads/sample and OTU counts. (XLSX)

## Abbreviations

FADES: Feeding and Autoimmunity in Down’s Syndrome Evaluation Study
DS: Down’s Syndrome
OUT: Operational Taxonomic Unit
