# Supplementary figures and images for: Gut microbiome analysis by post: Evaluation of the optimal method to collect stool samples from infants within a national cohort study
Source: PLoS One. 2019 Jun 12;14(6):e0216557. doi: 10.1371/journal.pone.0216557 (PMC6561628; doi:10.1371/journal.pone.0216557)

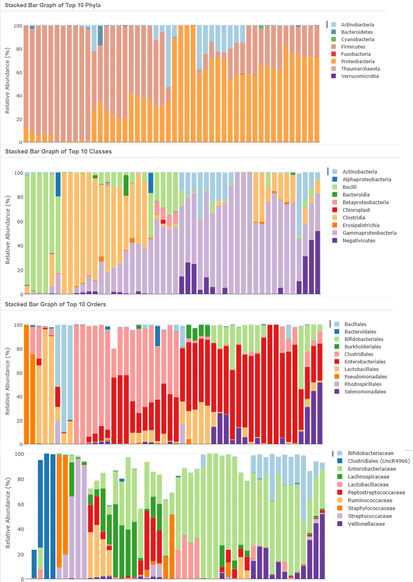

Supplement: S1 Fig — (TIF) [file pone.0216557.s001.tif]

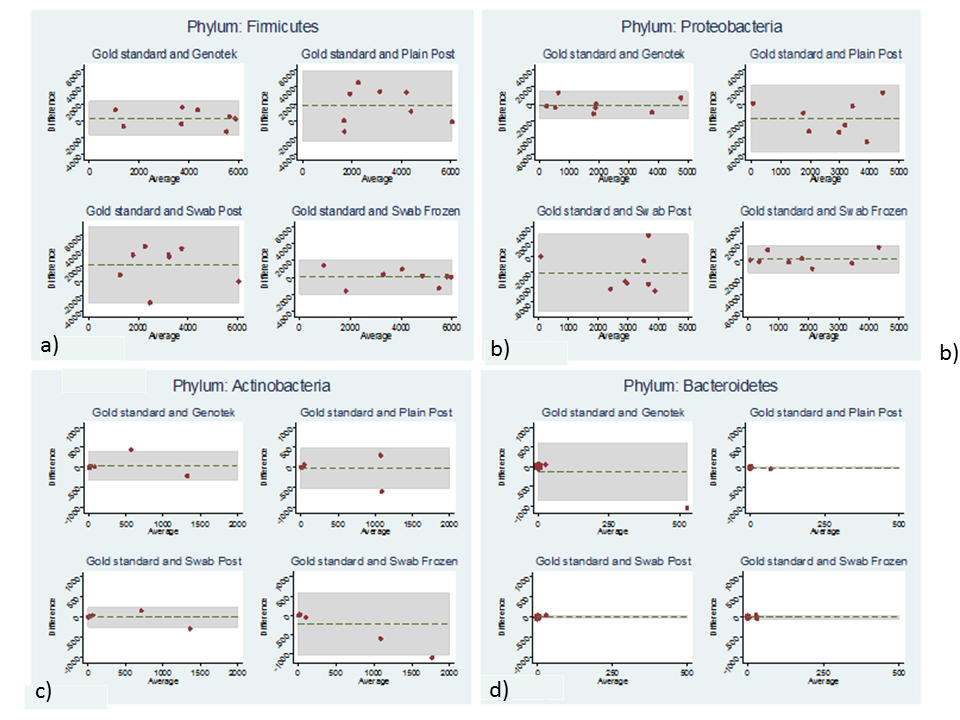

Supplement: S2 Fig — The Bland–Altman plots for abundance of a)Firmicutes, b)Proteobacteria, c)Actinobacteria and d)Bactriodetes for the four different methods of sample collection compared to the frozen standard of immediate freezing. (TIF) [file pone.0216557.s002.TIF]

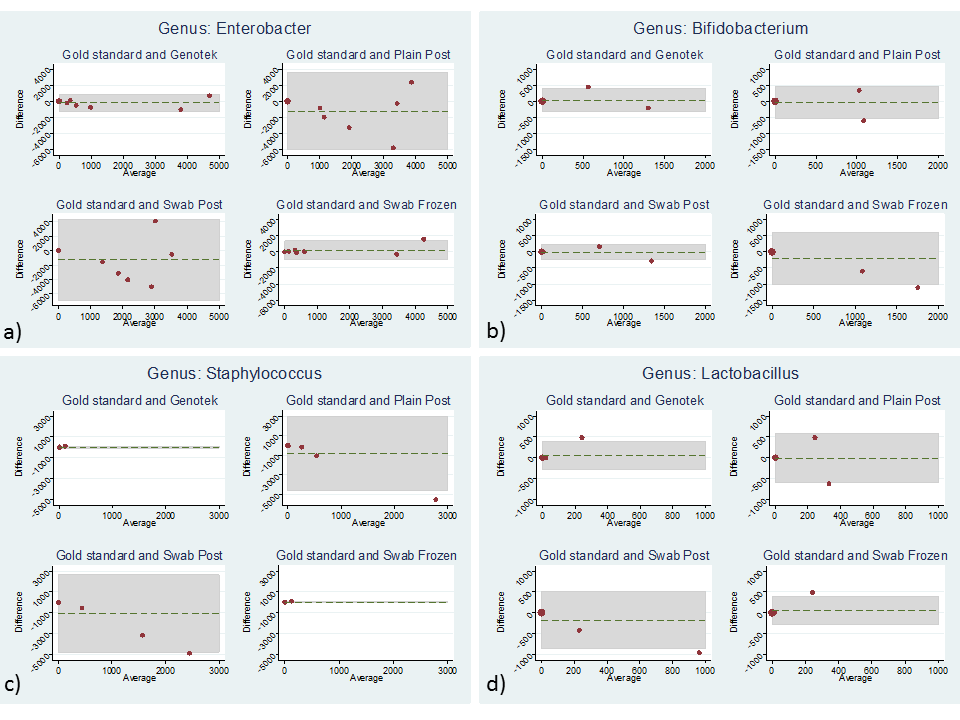

Supplement: S3 Fig — The Bland–Altman plots for abundance of a)Enterobacter, b)Bifidobacterium, c)Staphylococcus and d)Lactobacillus for the four different methods of sample collection compared to the frozen standard of immediate freezing. (TIF) [file pone.0216557.s003.TIF]
